# Targeted and Untargeted Proteomics-based Comparison of Adenoviral Infected hCMEC/D3 and hBMEC as a Human Brain Endothelial Cells to Study the OATP2B1 Transporter

**DOI:** 10.1007/s12035-025-04807-7

**Published:** 2025-03-14

**Authors:** Valerio Taggi, Anima M. Schäfer, Jonny Kinzi, Danilo Ritz, Isabell Seibert, Stefan Oswald, Henriette E. Meyer zu Schwabedissen

**Affiliations:** 1 Biopharmacy, Department of Pharmaceutical Sciences, University of Basel, Klingelbergstrasse 50, 4056 Basel, Switzerland; 2 Institute of Pharmacology and Toxicology, Rostock University Medical Center, Rostock, Germany; 3 Proteomics Core Facility, Biozentrum, University of Basel, Basel, Switzerland

**Keywords:** HCMEC/D3, HBMEC, OATP2B1, Adenoviral infection, Brain endothelial cells, Proteomics

## Abstract

The blood–brain barrier (BBB) is essential for central nervous system (CNS) homeostasis by regulating permeability between the bloodstream and brain. This study evaluates the immortalized human brain capillary endothelial cell lines hCMEC/D3 and hBMEC for their use as a brain endothelial cells to investigate the OATP2B1 transporter following adenoviral infection. We assessed the impact of adenoviral-mediated OATP2B1 expression on BBB marker proteins and transporters using targeted and untargeted mass spectrometry-based proteomics. Targeted proteomics identified measurable levels of endothelial markers PECAM1 and CDH5 in hCMEC/D3, whereas these markers were undetectable in hBMEC. Both cell lines exhibited similar Pgp levels, while BCRP was absent in hCMEC/D3. The expression of uptake transporters was also evaluated, revealing comparable levels of GLUT1, ENT1, MCT1 and OAT7 in hCMEC/D3 and hBMEC. Although OATP2B1 levels did not significantly increase post-infection in targeted proteomics, untargeted proteomics confirmed enhanced OATP2B1 expression. Other BBB markers and transporters remained unaffected in both cell lines. Notably, hCMEC/D3 demonstrated a stronger BBB phenotype, indicated by higher expression of BBB markers and transporters, while adenoviral infection was more effective in hBMEC. The differences between targeted and untargeted proteomics underscore the need for diverse methods to verify protein expression levels. This comparative analysis provides insights into the strengths and limitations of hCMEC/D3 and hBMEC for BBB research, particularly regarding drug transport mechanisms.

**Supplementary Information:**

The online version contains supplementary material available at 10.1007/s12035-025-04807-7.

## Introduction

The unique environment within the central nervous system (CNS) is crucial for ensuring normal neurological function. A structure that plays a major role in maintaining CNS homeostasis is the blood–brain barrier (BBB). Due to its highly selective permeability, the BBB governs the entrance of substances from the bloodstream into the brain [1]. The neurovascular unit composing the BBB consists of brain capillary endothelial cells (BCECs), pericytes, astrocytes, glial cells and neurons [2]. While each of these cell types plays a role in the overall function of the brain's microvasculature, BCECs are assumed to be responsible for the selective BBB permeability [3]. Recent proteomic studies have revealed significant age-related changes in the expression of various transporters at the BBB, including nutrient transporters such as SLC7A1 (CAT1), SLC7A5 (LAT1), and SLC59A1 (MFSD2A). These findings underscore the dynamic nature of the BBB transportome across different life stages and its potential impact on brain homeostasis and drug delivery [4]. Human immortalised BCECs are often used as in vitro models to study the BBB and its properties [5],Sivandzade & Cucullo, 2018). These models offer advantages, such as ease of use, reproducibility, and the ability to conduct high-throughput screenings. However, they also present limitations, namely the potential for genetic drift, de-differentiation and consequently an incomplete representation of in vivo BCECs characteristics [6]. Among the various cell lines employed, the human cell lines hCMEC/D3 and hBMEC have been used and characterised for their utility as a brain endothelial cells. When looking into the literature, greater attention was given to hCMEC/D3, which have been studied for their expression of tight junction proteins, surface receptors and transporters [1, 3, 7], revealing that they maintain the expression of various proteins normally found at the BBB. In addition, permeability of a range of test compounds varying in physicochemical properties and sizes were investigated in hCMEC/D3 [8]. hBMEC have also been evaluated, although less extensively. In particular, a study by Eigenmann et al*.* proposed hBMEC as a model to distinguish between permeable and non-permeable molecules at the BBB [9]. The two cell lines have also been compared for their use as brain endothelial cells, revealing a stronger BBB phenotype in hCMEC/D3 but higher resistance and lower permeability in hBMEC [10, 11].

An essential aspect of the BBB functionality is the presence of membrane transporters, which coordinate the flux of molecules across the BBB by controlling cellular efflux and uptake. More in detail, members of the ATP-binding cassette (ABC) family are responsible for the efflux of their substrates from the cells [12], whereas the organic anion-transporting polypeptides (OATPs) are involved in the uptake of drugs and endogenous compounds [13, 14]. Among OATPs, OATP2B1 is of particular interest, as it has been detected at the BBB in humans [15, 16] and it has been proposed to be involved in the uptake of statins and neurosteroids [14]. Despite that, its precise role remains unclear due to the lack of OATP2B1 expression at the BBB in animal models [17] and to limitations of human in vitro BBB models, which may not fully replicate the expression patterns of all BBB transporters [6, 11].

To address these limitations, methods such as adenoviral infection have been employed to transiently express specific proteins in cells, such as Hela, human coronary artery smooth muscle cells (HCASMC) and endothelial cells (HCAEC) models [18–20]. This approach allows the investigation of a specific transporter's function in the respective cellular environment. This approach allows the investigation of a specific transporter's function in the respective cellular environment. Of note, a first investigation of hCMEC/D3 and hBMEC as brain endothelial cells in which adenoviral expression of OATP2B1 was applied has already been performed in our research group [11]. In this study, Western blot analysis and transport experiments revealed higher expression and activity of OATP2B1 in hBMEC, respectively, while other features of the BBB cell model were not affected by adenoviral infection. An additional method to characterise the two cell lines and to evaluate the efficacy of the adenoviral infection in expressing protein of interest is the use of proteomics. More in detail, targeted proteomics provides absolute quantification of specific proteins, whereas untargeted proteomics offers a macroscopic analysis of the protein landscape, although in a more relative and less quantitative manner. As a consequence, combining the two approaches may enhance the information obtained and improve the reproducibility of the data [21, 22].

Accordingly, the aim of the herein described study was to further evaluate and characterise the Ad-OATP2B1 infected hCMEC/D3 and hBMEC for their use as a human brain endothelial cells to study the OATP2B1 transporter and to investigate the impact of the adenoviral infection on various proteins known to be of relevance in BCECs. MS-based targeted and untargeted proteomics were applied to hCMEC/D3 and hBMEC either infected with Ad-LacZ as control or Ad-OATP2B1 to test the transient expression of OATP2B1 and to assess the expression of key BBB markers, ABC and solute carrier (SLC) transporters in cells undergoing adenoviral infection.

## Materials and Methods

### Cell Culture

The brain microvascular endothelial cells hBMEC, generated by Professor Kwang Sik (Stins et al., 2001) and hCMEC/D3 (RRID:CVCL_U985) were kindly provided by Prof. Matthias Hamburger, Prof. Robin Teufel (Pharmaceutical Biology), and Prof. Jörg Huwyler (Pharmaceutical Technology) from the Department of Pharmaceutical Sciences, University of Basel, respectively. Both hBMEC and hCMEC/D3 are not authenticated cell lines. Nevertheless, the International Cell Line Authentication Committee has not indicated hBMEC or hCMEC/D3 as a misidentified cell line. Following the previous work conducted by Eigenmann et al*.* [10], hBMEC were cultured in EGM-2 basal medium supplemented with 20% Fetal Calf Serum (FCS, Thermo-Fisher Scientific, Karlsruhe, Germany), 1% penicillin–streptomycin (Thermo-Fisher Scientific) and SingleQuots kit composed of hydrocortisone, human basic fibroblast growth factor, vascular endothelial growth factor, human insulin like growth factor 1, ascorbic acid, epidermal growth factor, and heparin (CC-4176, Lonza, Basel, Switzerland). hCMEC/D3 were cultured in EGM-2 medium with the addition of 5% FCS, 1% penicillin–streptomycin, 1% HEPES (BioConcept AG, Allschwil, Switzerland), 1% Chemically Defined Lipid Concentrate™ (Thermo-Fisher Scientific), human basic fibroblast growth factor, ascorbic acid, and hydrocortisone (Sigma-Aldrich, Buchs, Switzerland) [11]. hBMEC used within the study were between passages 15 and 25, while hCMEC/D3 were between passages 28 and 35. Cells were grown at 37 °C in a humidified atmosphere with 5% CO_2_, and were cultured in rat-tail collagen type I (R&D Systems, Minneapolis, Minnesota) coated cultureware.

### Adenoviral Infection of hCMEC/D3 and hBMEC and Membrane Protein Sample Preparation

To analyse protein expression using targeted and untargeted proteomics, hCMEC/D3 and hBMEC were seeded in pre-coated 10 cm culture dishes (Sarstedt, Nümbrecht, Germany) at a density of 1,8 * 10^6^ cells/dish. 24 h later cells were infected with 200 plaque forming units (pfu) of either Ad-OATP2B1 or Ad-LacZ. The Ad-OATP2B1 was previously produced and characterised [19, 20], whereas the Ad-LacZ was provided by the ViraPower™ Adenoviral Expression System (Invitrogen, Carlsbad, CA, USA). 72 h after seeding, 100% confluent cells were harvested in ice-cold extraction buffer I (ProteoExtract® Native Membrane Protein Extraction Kit, Sigma-Aldrich) supplemented with 5 µl/ml of Protease Inhibitor Cocktail (PIC) (P8340, Sigma-Aldrich), transferred in LoBindTubes (Eppendorf, Hamburg, Germany) and incubated for 15 min at 4 °C while shaking at 100 rpm. Then, samples were centrifuged for 15 min at 16,000 g and 4 °C. The supernatant containing the cytoplasm protein was transferred in separated LoBindTubes, whereas the pellet was resuspended in extraction buffer II (ProteoExtract® Native Membrane Protein Extraction Kit, Sigma-Aldrich) supplemented with 5 µl/ml of PIC and incubated for 60 min at 4 °C while shaking at 100 rpm, followed by centrifugation for 15 min at 16,000 g and 4 °C. Subsequently, supernatant containing membrane proteins was collected in separated LoBindTubes, whereas the pellet was discarded. Finally, protein content was measured using the Pierce BCA Protein Assay Kit (Thermo Fisher Scientific) and the Microplate Reader Tecan Infinite M200 Pro (Tecan, Männedorf, Switzerland). Eight distinct cell culture preparations were made for targeted proteomics analysis, while three were prepared for untargeted proteomics analysis.

### LC–MS/MS Based Targeted Proteomics of hBMEC and hCMEC/D3

Following the isolation of membrane proteins, if necessary membrane fractions were adjusted to a maximum protein concentration of 2 mg/ml using ammonium bicarbonate buffer (50 mM, pH 7.8, Sigma-Aldrich). The lowest protein concentration used in this study was 0.066 mg/ml. Next, 50 µl of each membrane fraction were combined with 5 µl of dithiothreitol (200 mM, Sigma-Aldrich, Taufkirchen, Germany), 20 µl of ammonium bicarbonate buffer, and 5 µl of acetonitrile (Thermo Fisher Scientific) and incubated at 60 °C for 20 min. Cooling down the samples at room temperature (RT) was followed by the addition of 5 µl iodoacetamide (400 mM, Sigma-Aldrich) and a further incubation at 37 °C for 15 min. For protein digestion, 5 µl of trypsin (trypsin/protein ratio: 1/40, Promega, Mannheim, Germany) was added and the mixture was incubated at 37 °C for 16 h. Then, 10 µl of formic acid (10% v/v, Sigma-Aldrich) was added to stop the digestion process. The samples were subsequently centrifuged at 16,000 g and 4 °C for 15 min. From the resulting supernatant, 40 µl were taken and mixed with 40 µl of an isotope-labelled internal standard peptide mix (10 nM of each labelled peptide, Thermo Fisher Scientific). Analogous to a previously validated method [23], a 7500 QTRAP triple quadrupole mass spectrometer (AB Sciex, Darmstadt, Germany) coupled to an Agilent Technologies 1260 Infinity system (Agilent Technologies, Santa Clara, California) was used for protein quantification. Throughout the analysis, both precision (CV) and accuracy (error) were below 20%. The lower limit of quantification (LLOQ) was 0.04 nmol/L and values below LLOQ were excluded from the analysis. Final protein abundance data were normalised to the total protein amount of the isolated membrane fraction, as measured by the BCA assay. Thus, the protein-normalised LLOQ was 0.02 pmol/mg. Protein amount of PECAM1 (UniProtKB-ID: P16284), CDH5 (P33151), OCLN (Q16625), TFRC (P02786), GLUT1 (P11166), ENT1 (Q99808), Pgp (P08183), BCRP (Q9UNQ0), MRP1 (P33527), MRP4 (O15439), MCT1 (P53985), OAT2 (Q9Y694), OAT3 (Q8TCC7), OAT7 (Q8IVM8), OATP1A2 (P46721), OATP2B1 (O94956), OCT1 (O15245), OCT3 (O75751) and Na^+^/K^+^-ATPase (P05023) was quantified. The proteomic data have been submitted to the ProteomeXchange Consortium (https://www.proteomexchange.org/) through the MassIVE partner repository, using the MassIVE dataset identifier MSV000096107 and the ProteomeXchange identifier PXD056849. The targeted proteomics analysis via LC–MS/MS was funded by the German Research Foundation (project number: 505943254).

### Selection of Peptides and MRMs for Protein Analysis

Using in silico predictions, peptides specific for the proteins mentioned above were identified applying a method discussed elsewhere [24]*.* First, protein sequences taken from the UniProtKB/Swiss-Prot database were subjected to in silico trypsin digestion (www.expasy.org/tools)*,* leading to the generation of 7–25 amino acids peptide sequences, in which any missed cleavage site was excluded. Moreover, the following exclusion criteria were applied: peptides located in transmembrane regions, carrying N-terminal glutamic acid, methionine, cysteine or tryptophan, containing non-synonymous genetic polymorphisms with a frequency higher than 1%, or subjected to experimentally proven post-translational modifications. The specificity of each observed peptide was validated using an NCBI protein BLAST search against the UniProtKB/Swiss-Prot database. To establish the best multiple reaction monitoring (MRM) methods for the top peptides, the most suitable mass transitions were identified and fine-tuned. This was achieved by manually infusing synthetic peptides along with their stable isotope-labelled versions into a 7500 QTRAP triple quadrupole mass spectrometer (AB Sciex). For each peptide, the four mass transitions with the highest signal intensity were selected. Table 1 provides a summary of all proteotypic selected peptides and their optimised mass transitions.

**Table 1:** Overview of the selected proteotypic peptides and their corresponding mass transitions used in targeted proteomic analysis. The amino acid residue at the C-terminus (R or K) of the stable isotope-labelled peptide is marked with an asterisk

Protein	Peptide	Mass	Q1	z	Q3.1	Ion/z	Q3.2	Ion/z	Q3.3	Ion/z	Q3.4	Ion/z
PECAM1	STESYFIPEVR	1328.4	664.2	2 +	500.2	y4/1 +	760.3	y6/1 +	1010.4	y8/1 +	923.3	y7/1 +
	STESYFIPEVR*	1338.4	669.2	2 +	510.2	y4/1 +	770.3	y6/1 +	1020.4	y8/1 +	933.3	y7/1 +
CDH5	VDAETGDVFAIER	1422.5	711.2	2 +	1007.4	y9/1 +	906.3	y8/1 +	215.1	b2/1 +	1136.4	y10/1 +
	VDAETGDVFAIER*	1432.5	716.2	2 +	1017.4	y9/1 +	916.3	y8/1 +	215.1	b2/1 +	1146.5	y10/1 +
OCLN	YSSGGNFETPSK	1274.3	637.2	2 +	1023.3	y10/1 +	331.2	y3/1 +	1110.3	y11/1 +	936.3	y9/1 +
	YSSGGNFETPSK*	1282.3	641.2	2 +	1031.3	y10/1 +	339.2	y3/1 +	1118.3	y11/1 +	944.3	y9/1 +
TFRC	LAVDEEENADNNTK	1562.6	781.2	2 +	185.1	b2/1 +	905.3	y8 + 1	1034.3	y9/1 +	1163.3	y10/1 +
	LAVDEEENADNNTK*	1570.6	785.1	2 +	185.1	b2/1 +	913.3	y8 + 2	1042.3	y9/1 +	1171.2	y10/1 +
GLUT1	TFDEIASGFR	1143.2	571.7	2 +	894.3	y8/1 +	537.2	y5/1 +	650.2	y6/1 +	779.3	y7/1 +
	TFDEIASGFR*	1153.2	576.7	2 +	904.4	y8/1 +	547.2	y5/1 +	660.2	y6/1 +	789.3	y7/1 +
ENT1	LEGPGEQETK	1088.2	544.2	2 +	845.2	y8/1 +	243.1	b2/1 +	691.2	y6/1 +	788.2	y7/1 +
	LEGPGEQETK*	1096.2	548.2	2 +	853.3	y8/1 +	243.1	b2/1 +	699.2	y6/1 +	796.2	y7/1 +
Pgp	AGAVAEEVLAAIR	1270.5	635.5	2 +	971.5	y9/1 +	900.5	y8/1 +	430.2	y4/1 +	771.5	y7/1 +
	AGAVAEEVLAAIR*	1280.5	640.5	2 +	981.5	y9/1 +	910.5	y8/1 +	440.2	y4/1 +	781.4	y7/1 +
BCRP	SSLLDVLAAR	1044.6	522.9	2 +	644.3	y6/1 +	757.5	y7/1 +	529.3	y5/1 +	430.3	y4/1 +
	SSLLDVLAAR*	1054.6	527.9	2 +	654.4	y6/1 +	767.5	y7/1 +	539.3	y5/1 +	440.3	y4/1 +
MRP1	DGAFAEFLR	1026.1	513.4	2 +	635.2	y5/1 +	782.2	y6/1 +	853.0	y7/1 +	564.1	y4/1 +
	DGAFAEFLR*	1036.1	518.4	2 +	644.9	y5/1 +	792.2	y6/1 +	863.2	y7/1 +	574.4	y4/1 +
MRP4	SSLISALFR	994.2	497.0	2 +	593.1	y5/1 +	706.3	y6/1 +	819.5	y7/1 +	506.3	y4/1 +
	SSLISALFR*	1004.2	502.4	2 +	603.2	y5/1 +	716.3	y6/1 +	829.4	y7/1 +	516.2	y4/1 +
MCT1	DLHDANTDLIGR	1340.4	447.1	3 +	345.2	y3/1 +	458.2	y4/1 +	232.1	y2/1 +	573.2	y5/1 +
	DLHDANTDLIGR*	1350.4	450.4	3 +	355.2	y3/1 +	468.2	y4/1 +	242.1	y2/1 +	583.2	y5/1 +
OAT2	NVALLALPR	967.2	483.8	2 +	753.4	y7/1 +	569.3	y5/1 +	682.3	y6/1 +	214.1	b2/1 +
	NVALLALPR*	977.2	488.8	2 +	763.4	y7/1 +	214.1	y5/1 +	579.1	y6/1 +	692.4	b2/1 +
OAT3	VAVFNGK	734.4	367.7	2 +	564.2	y5/1 +	635.2	y6/1 +	465.1	y4/1 +	171.1	b2/1 +
	VAVFNGK*	741.4	371.7	2 +	572.2	y5/1 +	643.2	y6/1 +	473.2	y4/1 +	171.1	b2/1 +
OAT7	NKPLFDTIQDEK	1448.6	724.2	2 +	243.0	b2/1 +	603.3	b5/1 +	995.2	y8/1 +	715.3	b6/1 +
	NKPLFDTIQDEK*	1456.6	728.0	2 +	243.1	b2/1 +	607.2	b5/1 +	1003.2	y8/1 +	719.6	b6/1 +
OATP1A2	EGLETNADIIK	1203.3	602.0	2 +	774.3	y7/2 +	673.3	y6/1 +	903.1	y8/1 +	559.3	y5/1 +
	EGLETNADIIK*	1213.3	606.9	2 +	783.5	y7/2 +	681.0	y6/1 +	912.4	y8/1 +	567.5	y5/1 +
OATP2B1	SSPAVEQQLLVSGPGK	1596.8	799.2	2 +	712.0	y14/2 +	445.2	y5/1 +	1155.6	y11/1 +	544.2	y6/1 +
	SSPAVEQQLLVSGPGK*	1604.8	803.0	2 +	716.0	y14/2 +	453.3	y5/1 +	1163.7	y11/1 +	552.3	y6/1 +
OATP2B1	YYNNDLLR	1070.5	535.8	2 +	744.3	y6/1 +	327.1	b2/1 +	907.3	y7/1 +	630.3	y5/1 +
	YYNNDLLR*	1080.5	540.8	2 +	754.3	y6/1 +	327.1	b2/1 +	917.3	y7/1 +	640.3	y5/1 +
OCT1	ENTIYLK	881.0	440.9	2 +	637.2	y5/1 +	536.3	y4/1 +	423.1	y3/1 +	260.1	y2/1 +
	ENTIYLK*	889.0	444.9	2 +	645.3	y5/1 +	544.3	y4/1 +	431.2	y3/1 +	268.1	y2/1 +
OCT3	GIALPETVDDVEK	1386.6	693.5	2 +	1031.3	y9/1 +	355.1	b4/1 +	242.1	b3/1 +	171.1	b2/1 +
	GIALPETVDDVEK*	1394.6	697.5	2 +	1039.4	y9/1 +	242.1	b4/1 +	355.1	b3/1 +	171.1	b2/1 +
Na^+^/K^+^-ATPase	LSLDELHR	983.1	491.8	2 +	669.3	y5/1 +	782.4	y6/1 +	869.4	y7/1 +	554.3	y4/1 +
	LSLDELHR*	993.1	496.8	2 +	679.3	y5/1 +	792.4	y6/1 +	879.4	y7/1 +	564.3	y4/1 +

### hBMEC and hCMEC/D3 Membrane Protein Digest and Untargeted Proteomics Analysis

Subsequent to the isolation of membrane proteins, samples were resuspended in 5% SDS, 10 mM Tris(2-carboxyethyl)phosphine hydrochloride (TCEP, Sigma-Aldrich), 0.1 M Triethylammonium bicarbonate (TEAB) and incubated for 10 min at 95 °C while shaking at 500 rpm. Proteins were alkylated in 20 mM iodoacetamide for 30 min at 25 °C and digested using S-Trap™ micro spin columns (Protifi, New York, USA) according to the manufacturer’s instructions. Shortly, 12% phosphoric acid was added to each sample (final concentration of phosphoric acid 1.2%) followed by the addition of S-trap buffer (90% methanol, 100 mM TEAB pH 7.1) at a ratio of 6:1. Samples were mixed by vortexing and loaded onto S-trap columns by centrifugation at 4000 g for 1 min followed by three washes with S-trap buffer. Digestion buffer (50 mM TEAB pH 8.0) containing sequencing-grade modified trypsin (Promega) was added to the S-trap column and samples were incubated for 1 h at 47 °C. Peptides were eluted by the consecutive addition and collection by centrifugation at 4000 g for 1 min of 40 ul digestion buffer, 40 ul of 0.2% formic acid and finally 35 ul 50% acetonitrile, 0.2% formic acid. Samples were dried under vacuum and stored at −20 °C. The day of the analysis, dried peptides were resuspended in 0.1% aqueous formic acid, 0.02% DDM (n-Dodecyl-B-D-maltoside) and subjected to LC–MS/MS analysis using a timsTOF Ultra Mass Spectrometer (Bruker, Fällanden, Switzerland) equipped with a CaptiveSpray nano-electrospray ion source (Bruker) and fitted with a Vanquish Neo (Thermo Fisher Scientific). Peptides were resolved using a RP-HPLC column (100 µm × 30 cm) packed in-house with C18 resin (ReproSil Saphir 100 C18, 1.5 µm resin; Dr. Maisch GmbH, Ammerbuch, Germany) at a flow rate of 0.4 µl/min and column heater set to 60 °C. The following gradient was used for peptide separation: from 2% B to 25% B over 25 min to 35% B over 5 min to 95% B over 1 min followed by 5 min at 95% B to 2% B over 1 min followed by 3 min at 2% B. Buffer A was 0.1% formic acid in water and buffer B was 80% acetonitrile, 0.1% formic acid in water. The mass spectrometer was operated in dia-PASEF mode with a cycle time estimate of 0.95 s. MS1 and MS2 scans were acquired over a mass range from 100 to 1700 m/z. A method with 8 dia-PASEF scans separated into 3 ion mobility windows per scan covering a 400–1000 m/z range with 25 Da windows and an ion mobility range from 0.64 to 1.37 Vs cm^2^ was used. Accumulation and ramp time were set to 100 ms, capillary voltage was set to 1600 V, dry gas was set to 3 l/min and dry temperature was set to 200 °C. The collision energy was ramped linearly as a function of ion mobility from 59 eV at 1/K0 = 1.6 V s cm^−2^ to 20 eV at 1/K0 = 0.6 V s cm^−2^. The acquired files were searched using the Spectronaut (Biognosys v18.6, Schlieren, Switzerland) directDIA workflow against a Homo sapiens database (consisting of 20,360 protein sequences downloaded from Uniprot on 20,220,222) and 393 commonly observed contaminants using default settings. Proteomic data have been deposited to the ProteomeXchange Consortium (https://www.proteomexchange.org/) via the MassIVE partner repository with MassIVE data set identifier MSV000096107 and ProteomeXchange identifier PXD056849.

### Statistical Analysis

The targeted proteomics data were analysed using GraphPad Prism version 10.2 (GraphPad Software, La Jolla, California). Statistical significance was set at a P-value of ≤ 0.05. Data were assessed for normality applying the Shapiro–Wilk test. One-way ANOVA followed by Tukey’s post-hoc test for multiple comparisons or unpaired t-test for single comparison were conducted. For non-normal data, Brown-Forsythe and Welch's ANOVA was used for multiple comparisons, and Mann–Whitney test was used for single comparison. No outlier tests were performed. For untargeted proteomics analysis, quantitative fragment ion data (F.Area) was exported from Spectronaut and analysed using the MSstats R package v.4.9.9. (10.1093/bioinformatics/btu305). Qvalue filter of 0.01 was applied during data import. Data was normalised using the default normalisation option “equalizedMedians”, imputed using “AFT model-based imputation” and P-values for pairwise comparisons were calculated as implemented in MSstats. To identify expression differences, we defined our selection criteria for differentially expressed proteins as a minimum twofold change with statistical significance (*P*-value < 0.05).

## Results

### Absolute Quantification of BBB Markers in Adenoviral Infected hCMEC/D3 and hBMEC

**Fig. 1:**
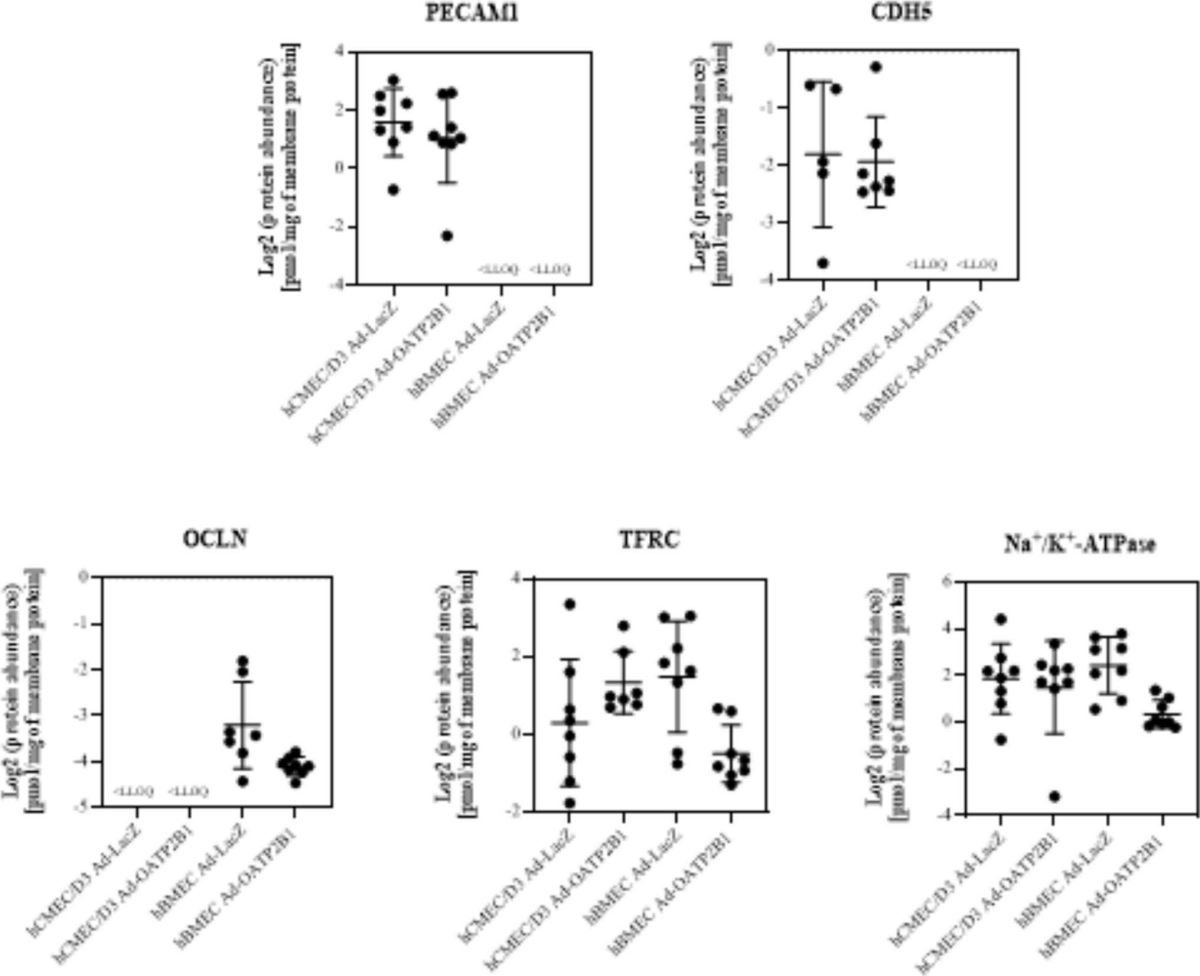
Comparison of BBB markers expression in hCMEC/D3 Ad-LacZ, hBMEC Ad-LacZ, hCMEC/D3 Ad-OATP2B1 and hBMEC Ad-OATP2B1. PECAM1, CDH5, OCLN, TFRC and Na^+^/K^+^-ATPase protein abundance [pmol/mg of membrane protein] in hCMEC/D3 and hBMEC infected with either Ad-LacZ or Ad-OATP2B1. One-way ANOVA followed by Tukey's test for multiple comparisons, Brown-Forsythe and Welch's ANOVA, or unpaired t-test or Mann–Whitney test were used. n = 3–8. Samples below the lower limit of quantification (LLOQ) were excluded from the analysis. Results are reported as mean ± SD. Images created with GraphPad Prism version 10.2

An important feature of a BBB cell model is the presence of endothelial proteins, tight junctions and receptors [5]. Following a previous study, where we reported on the expression and functionality of OATP2B1 in adenoviral infected hCMEC/D3 and hBMEC [11], we conducted targeted proteomics in hCMEC/D3 Ad-LacZ, hBMEC Ad-LacZ, hCMEC/D3 Ad-OATP2B1, and hBMEC Ad-OATP2B1, comparing the expression of BBB markers between the two cell lines and evaluating the effects of the adenoviral infection on their expression. As shown in Fig. 1, the endothelial markers PECAM1 and CDH5 were detected only in hCMEC/D3, in which the protein abundance was found to be comparable between Ad-LacZ and Ad-OATP2B1 infected cells. Conversely, although not affected by the adenoviral infection, the protein level of the tight junction OCLN was above the lower limit of quantification (LLOQ) only in hBMEC. Finally, protein expression of the BBB receptor TFRC was measurable and comparable in both hCMEC/D3 and hBMEC independently from the adenoviral infection. As control, Na^+^/K^+^-ATPase protein abundance was also evaluated. Table 2 reports the values below LLOQ measured in the two cell lines for the above mentioned BBB markers.

**Table 2:** BBB markers under limit of quantification. Absolute protein abundance below lower limit of quantification (LLOQ) of PECAM1 and CDH5 in hBMEC Ad-LacZ and hBMEC Ad-OATP2B1 and of OCLN in hCMEC/D3 Ad-LacZ and hCMEC/D3 Ad-OATP2B1. Values are expressed as pmol/ml. Results are reported as mean ± SD of n = 8

	< LLOQ [pmol/ml]		
	PECAM1	CDH5	OCLN
hCMEC/D3 Ad-LacZ	> LLOQ	> LLOQ	0.034 ± 0.026
hBMEC Ad-LacZ	0.018 ± 0.007	0.010 ± 0.003	> LLOQ
hCMEC/D3 Ad-OATP2B1	> LLOQ	> LLOQ	0.026 ± 0.022
hBMEC Ad-OATP2B1	0.023 ± 0.003	0.016 ± 0.007	> LLOQ

### Targeted Proteomics Quantification of Efflux Transporters in hCMEC/D3 Ad-LacZ, hBMEC Ad-LacZ, hCMEC/D3 Ad-OATP2B1 and hBMEC Ad-OATP2B1

**Fig. 2:**
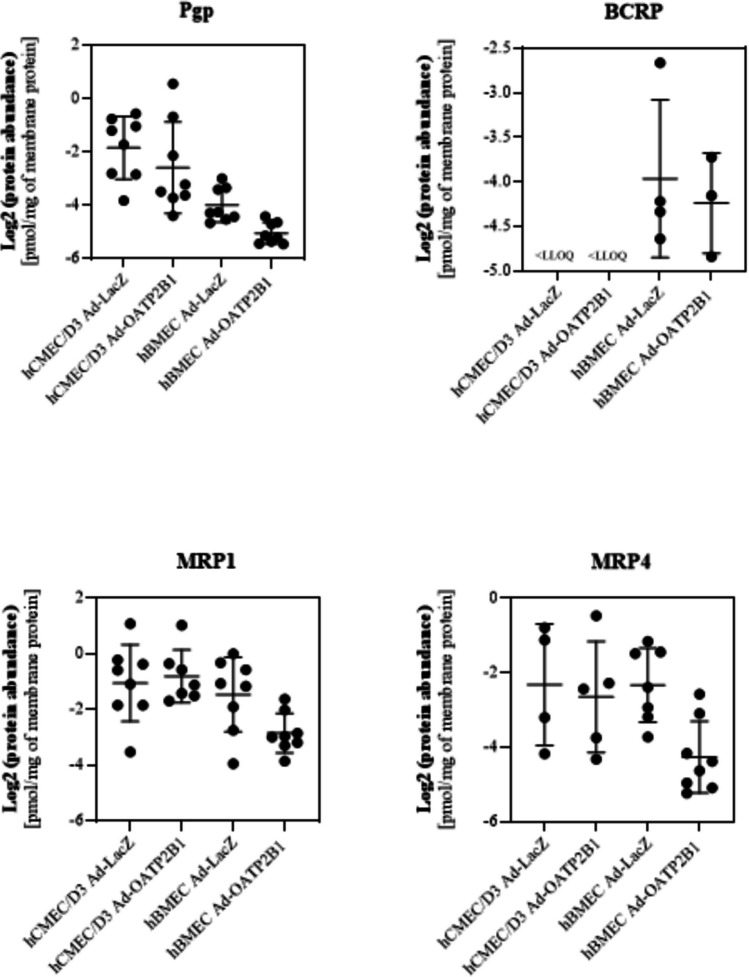
Evaluation of efflux transporters in hCMEC/D3 Ad-LacZ, hBMEC Ad-LacZ, hCMEC/D3 Ad-OATP2B1 and hBMEC Ad-OATP2B1. Protein level of Pgp, BCRP, MRP1 and MRP4 was quantified as pmol/mg of membrane protein in the differently infected hCMEC/D3 and hBMEC. One-way ANOVA followed by Tukey's test for multiple comparisons, Brown-Forsythe and Welch's ANOVA, unpaired t-test or Mann–Whitney test were used. *n* = 3–8. Samples below LLOQ were excluded from the analysis. Data are shown as mean ± SD. Images created with GraphPad Prism version 10.2

A key feature of a robust in vitro BBB cell model is the expression of efflux transporters, as these proteins play an important role in the selective permeability that characterises the BBB. Among them, Pgp, BCRP and multidrug resistance proteins (MRPs) are prominently expressed at the BBB [25]. Accordingly, following the quantification of BBB endothelial, tight junction and receptor proteins, we wanted to compare the two cell lines for their protein level of efflux transporters and to verify that the adenoviral induced expression of OATP2B1 did not modify their expression levels. As displayed in Fig. 2, targeted proteomics analysis showed that the abundance of Pgp was comparable between hCMEC/D3 and hBMEC independently from the adenoviral infection. Conversely, BCRP expression was measurable in hBMEC Ad-LacZ and hBMEC Ad-OATP2B1 only, whereas hCMEC/D3 Ad-LacZ and hCMEC/D3 Ad-OATP2B1 levels were found to be below LLOQ (Fig. 2 and Table 3). Finally, the protein quantification of MRPs revealed that MRP1 and MRP4 protein abundance was comparable between hCMEC/D3 Ad-LacZ, hBMEC Ad-LacZ, hCMEC/D3 Ad-OATP2B1, and hBMEC Ad-OATP2B1 (Fig. 2).

**Table 3:** BCRP protein expression in hCMEC/D3 Ad-LacZ and hCMEC/D3 Ad-OATP2B1. Below LLOQ absolute protein abundance of BCRP [pmol/ml] in hCMEC/D3 and hCMEC/D3 Ad-OATP2B1. Results are shown as mean ± SD of n = 8

	< LLOQ [pmol/ml]
	BCRP
hCMEC/D3 Ad-LacZ	0.020 ± 0.008
hBMEC Ad-LacZ	> LLOQ
hCMEC/D3 Ad-OATP2B1	0.035 ± 0.012
hBMEC Ad-OATP2B1	> LLOQ

### Targeted Proteomics Analysis of SLC Transporters Following Adenoviral Infection in hCMEC/D3 and hBMEC

**Fig. 3:**
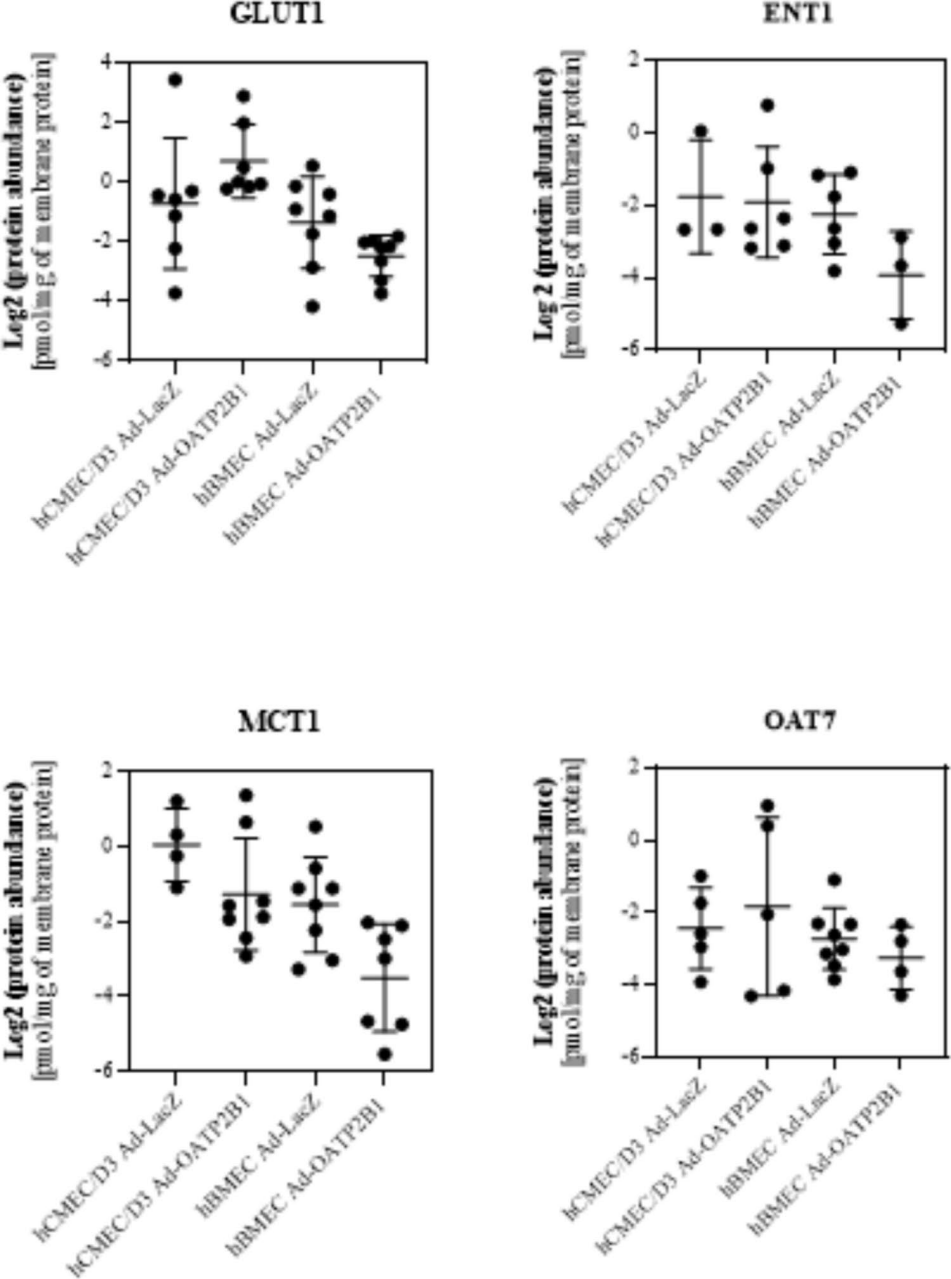
Uptake transporters expression in hCMEC/D3 Ad-LacZ, hBMEC Ad-LacZ, hCMEC/D3 Ad-OATP2B1 and hBMEC Ad-OATP2B1. GLUT1, ENT1, MCT1, and OAT7 protein levels [pmol/mg of membrane protein] were quantified in Ad-LacZ and Ad-OATP2B1 infected hCMEC/D3 and hBMEC. One-way ANOVA followed by Tukey's test for multiple comparisons, Brown-Forsythe and Welch's ANOVA, or unpaired t-test or Mann–Whitney test were used. Data from samples below the LLOQ were excluded from the analysis. Results are presented as mean ± SD, with sample sizes ranging from n = 3 to n = 8. Images created with GraphPad Prism version 10.2

Apart from OATP2B1, other SLC transporters are essential at the BBB for the permeation of compounds that play a role in the physiological function of the brain, such as glucose, nucleosides, thyroid hormones and neurosteroids [26, 27]. Consequently, to broaden the comparison of the two cell lines for their applicability as a BBB cell model and to confirm that the Ad-OATP2B1 infection led to a selectively enhanced expression of OATP2B1, we also evaluated the protein level of other SLC transporters with a major role at the BBB. As shown in Fig. 3, the expression of GLUT1 and ENT1 was comparable among hCMEC/D3 Ad-LacZ, hBMEC Ad-LacZ, hCMEC/D3 Ad-OATP2B1 and hBMEC Ad-OATP2B1. Similar results were found for MCT1, whose protein abundance did not differ in the two brain endothelial cell lines (Fig. 3 and Table 4). When focusing on OATs and OCTs, diverse results emerged. OAT7 abundance was similar between hCMEC/D3 and hBMEC, regardless of adenoviral infection (Fig. 3). In contrast, the protein levels of OAT2, OAT3, OCT1, and OCT3 were either below LLOQ or undetectable. Similarly, OATP1A2 was found to be below LLOQ or not detected in both cell lines, independent of infection status (Table 4). As one of the main goals of this study was to compare the expression of the OATP2B1 transporter in hCMEC/D3 and hBMEC following Ad-OATP2B1 infection, two distinct peptides—SSPAVEQQLLVSGPGK and YYNNDLLR—were used for quantification. Despite previous confirmation of increased OATP2B1 expression in both cell lines after adenoviral infection via Western blot analysis [11], OATP2B1 abundance for both peptides was found to be below LLOQ in hCMEC/D3 and hBMEC, regardless of adenoviral infection (Table 4).

**Table 4:** SLC transporters below LLOQ in hCMEC/D3 Ad-LacZ, hBMEC Ad-LacZ, hCMEC/D3 Ad-OATP2B1 and hBMEC Ad-OATP2B1. Absolute protein abundance of OAT2, OAT3, OCT1, OCT3, OATP1A2, and OATP2B1 below LLOQ in hCMEC/D3 and hBMEC either infected with Ad-LacZ or Ad-OATP2B1. The data are expressed as mean ± SD in pmol/ml, with a total sample size of n = 8. ND = not detected

	< LLOQ [pmol/ml]						
	OAT2	OAT3	OCT1	OCT3	OATP1A2	OATP2B1	OATP2B1
						SSPAVEQQLLVSGPGK	YYNNDLLR
hCMEC/D3 Ad-LacZ	0.020 ± 0.010	ND	ND	ND	0.001 ± 0.001	0.008 ± 0.007	0.004 ± 0.005
hBMEC Ad-LacZ	0.019 ± 0.010	ND	ND	ND	0.009 ± 0.009	0.009 ± 0.008	0.019 ± 0.020
hCMEC/D3 Ad-OATP2B1	0.017 ± 0.004	ND	ND	ND	ND	0.004 ± 0.005	0.011 ± 0.018
hBMEC Ad-OATP2B1	0.024 ± 0.007	ND	ND	ND	0.004 ± 0.006	0.019 ± 0.020	0.043 ± 0.048

### Untargeted Proteomics Analysis of hCMEC/D3 and hBMEC Infected with Ad-LacZ and Ad-OATP2B1

**Fig. 4:**
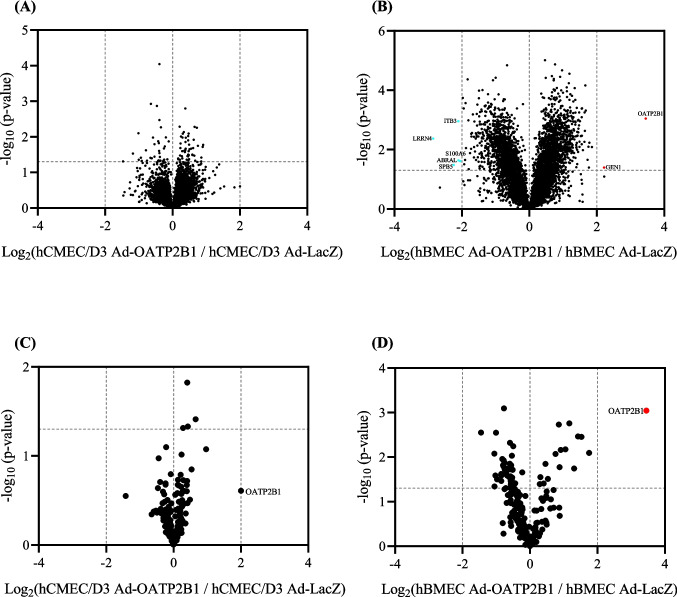
Volcano Plots showing the relative expression of membrane proteins in adenovirus-infected hCMEC/D3 and hBMEC. Relative membrane protein expression in hCMEC/D3 Ad-OATP2B1 in respect to hCMEC/D3 Ad-LacZ (**A**) and hBMEC Ad-OATP2B1 in respect to hBMEC Ad-LacZ (**B**), with a focus on BBB markers, ABC and SLC transporters (**C**-**D**). Unpaired t-test was used for statistical analysis. Red or blue dots show protein with an at least twofold statistically significant difference (*P*-value ≤ 0.05) in expression. Three distinct cell culture preparations were performed. Triplicates were averaged. Images created with GraphPad Prism version 10.2

An effective way to describe and compare hCMEC/D3 and hBMEC is the use of untargeted proteomics analysis. While this approach does not quantify absolute protein levels, it provides a broad characterization of the two cell lines. At first, we wanted to verify the efficacy of the Ad-OATP2B1 infection in selectively increasing the expression of the transporter without modifying the level of other key BBB proteins. Consequently, we performed a comparison within the same cell line but between Ad-LacZ and Ad-OATP2B1 infected cells. As reported in Supplementary Table 1, we identified 6690 proteins in hCMEC/D3 and 6860 proteins in hBMEC. A generally lower variation in protein level and no protein with a biologically meaningful different expression level was found in hCMEC/D3 Ad-LacZ and hCMEC/D3 Ad-OATP2B1, whereas a higher variation in expression could be observed when comparing hBMEC Ad-LacZ and hBMEC Ad-OATP2B1, mainly for proteins related to the cell cycle, the adenoviral infection and the subsequent DNA modification (Fig. 4A and B). Specifically, the level of GEN1 (involved in Holliday junction resolution, DNA repair, meiosis and regulation of the cell cycle [28–30]) was increased in hBMEC Ad-OATP2B1, whereas in hBMEC Ad-LacZ enhanced protein expression was found for ITB3 (partaking in cell signalling [31, 32]), LRRN4 (contributing to cell adhesion and signal transduction [33, 34]), ABRAL (active in cell migration [35]), S100A6 (associated with cell proliferation and differentiation [36]), and SPB5 (connected to cell signalling [37]). In addition, the analysis revealed that the Ad-OATP2B1 infection increased the expression of OATP2B1 in both hCMEC/D3 and hBMEC but did not modify the expression of BBB markers, ABC and SLC transporters (Fig. 4C and D). Importantly, although OATP2B1 protein level was enhanced in both cell lines, the difference in OATP2B1 level between Ad-LacZ infected and Ad-OATP2B1 infected cells was statistically significant in hBMEC only. The list of selected BBB markers, ABC and SLC transporters is reported in Supplementary Table 2.

### Comparison of Ad-OATP2B1 Infected hCMEC/D3 and hBMEC by Untargeted Proteomics

**Fig. 5:**
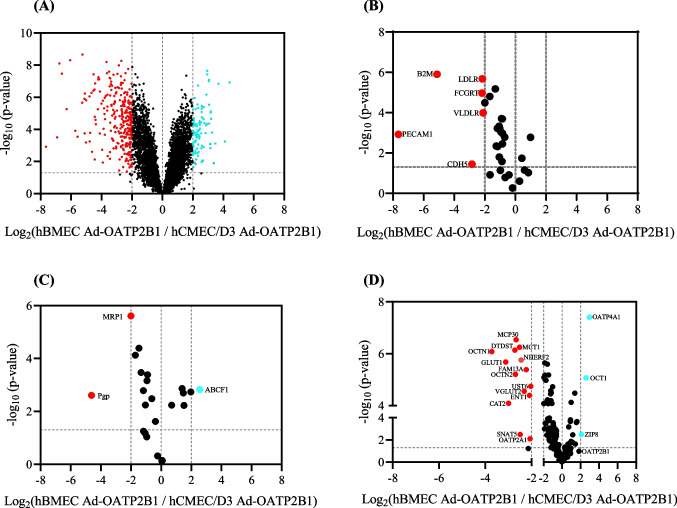
Volcano plots displaying the relative expression of membrane proteins in hBMEC versus hCMEC/D3. Comparative membrane protein expression in hBMEC Ad-OATP2B1 in respect to hCMEC/D3 Ad-OATP2B1 (**A**). Emphasis was given to BBB markers (**B**), ABC transporters (**C**), and SLC transporters (**D**). Statistical analysis was performed using an unpaired t-test. Proteins with at least a twofold statistically significant difference (*P*-value ≤ 0.05) in expression are indicated by red or blue dots. Three individual cell culture preparations were conducted. Data from the replicates were averaged. Images created with GraphPad Prism version 10.2

After confirming that Ad-OATP2B1 infection increased OATP2B1 expression without affecting the protein levels of key BBB markers, we proceeded with comparing hCMEC/D3 Ad-OATP2B1 and hBMEC Ad-OATP2B1. One major goal of this study is to evaluate these two cell lines as a human in vitro model for studying OATP2B1 at the BBB. Although the analysis revealed 6498 shared proteins (Supplementary Table 1), the expression of several proteins differed between hBMEC Ad-OATP2B1 and hCMEC/D3 Ad-OATP2B1 (Fig. 5A). More in detail, a thorough assessment of the two cell lines displayed that hCMEC/D3 Ad-OATP2B1 had a higher protein expression of the BBB markers B2M, PECAM1, CDH5, FCGRT, LDLR and VLDLR (Fig. 5B). Furthermore, hCMEC/D3 Ad-OATP2B1 showed enhanced protein level of the ABC transporters Pgp and MRP1, whereas hBMEC Ad-OATP2B1 had an augmented level of ABC50 (Fig. 5C). Finally, when observing the expression of SLC transporters, higher expression was observed for various SLC transporters in hCMEC/D3 Ad-OATP2B1, whereas only OATP4A1, S22AI and ZIP8 were elevated in hBMEC Ad-OATP2B1 (Fig. 5D). Of note, although not statistically significant, OATP2B1 expression was higher in hBMEC Ad-OATP2B1 compared to hCMEC/D3 Ad-OATP2B1.

## Discussion

In this study, we characterised and compared the applicability of Ad-OATP2B1 infected hCMEC/D3 and hBMEC as a human in vitro brain endothelial cell model to study the OATP2B1 transporter. For this, we determined the change in OATP2B1 expression after adenoviral gene transfer in the two cell lines and the influence of the transporter on the expression of key BBB proteins.

From our perspective, a robust brain endothelial cell model should express a broad spectrum of proteins normally found at the BBB in vivo [5]. At first, applying targeted proteomics we assessed whether the Ad-OATP2B1 infection would affect the expression of 17 proteins (compare Table 1) selected for this characteristic. Among these are BBB markers and transporters, none of which showed a change in abundance when comparing Ad-LacZ and Ad-OATP2B1 cells neither in hCMEC/D3, nor in hBMEC. In the next step, we compared the results obtained by targeted proteomics in samples from hCMEC/D3 and hBMEC. Regardless of the adenoviral infection, TFRC expression was comparable between the two cell lines. However, hCMEC/D3 cells showed a higher abundance of the endothelial markers PECAM1 and CDH5, while their expression in hBMEC was below the lower limit of quantification (LLOQ). These results are in line with a previous report, in which the expression of BBB markers was evaluated performing real-time quantitative PCR and Western blot analysis [11]. Conversely, the expression of the tight junction protein OCLN was above LLOQ only in hBMEC. As OCLN is involved in the control of permeability [11], its higher expression in hBMEC may explain the previously observed greater cell layer tightness when quantified in cells cultured on transwells through the assessment the transendothelial electrical resistance (TEER), the electrical capacitance (C_CL_), and the cellular permeability [10, 11].

Besides BBB markers, membrane transporters are critical for maintaining the selective BBB permeability of the BBB and play a key role in the pharmacokinetics of drugs at this structure [26, 38]. In contrast to what we showed in our previous study [11], targeted proteomics revealed comparable levels of the efflux transporter Pgp in hCMEC/D3 and hBMEC. However, BCRP was detected in hBMEC but was found to be below LLOQ in Ad-lacZ and Ad-OATP2B1 hCMEC/D3. These results are in opposition to previous literature, which demonstrated the expression and function of BCRP [11, 39]. We also compared the levels of uptake transporters, observing comparable levels of GLUT1, ENT1, MCT1, and OAT7 in hCMEC/D3 and hBMEC, independent of the adenoviral infection. However, the expression of the uptake transporters OAT2, OAT3, OCT1, OCT3, and OATP1A2, which have also been reported to be expressed at the BBB [15, 40, 41], was below the LLOQ in both cell lines regardless of the adenoviral infection.

To contextualize our results in hCMEC/D3 and hBMEC, we compared our findings with targeted proteomics data from human BBB samples reported in the literature [41]. This evaluation revealed that protein levels of the herein investigated transporters and receptors were generally lower in samples from hCMEC/D3 and hBMEC cells than in those from human brain microvessels. However, we cannot comment on the expression levels of BBB markers, as, to our knowledge, no proteomics data are available on their levels in human brain microvessels.

One potential explanation for the lack of protein detection observed performing targeted proteomics could be the low protein concentrations in the membrane protein fraction prepared, particularly from hCMEC/D3 cells. Indeed, if protein concentrations are low, the resulting samples may not provide sufficient material for accurate detection. Additionally, the subsequent dilution that occurs during the analysis by the LC–MS/MS device (7500 QTRAP triple quadrupole mass spectrometer (AB Sciex, Darmstadt, Germany) coupled to an Agilent Technologies 1260 Infinity system) may further decrease the respective peptide concentration in the sample, making accurate quantification even more challenging [42–44]. Thus, both the initial low protein concentration and the dilution occurred during the analysis likely contribute to the sub-LLOQ levels observed for some proteins in the targeted proteomics analysis. Moreover, it is unclear whether the obtained results were affected by the fact that we did not employ additional detergents and solubilizers during peptide preparation. More in detail, we applied the ProteoExtract® Native Membrane Protein Extraction Kit from Sigma-Aldrich, which is designed to efficiently extract membrane proteins while maintaining their native conformations. The proprietary reagents in the kit are optimized to solubilize membrane proteins effectively without the need for additional detergents or solubilizers prior to reduction, alkylation, and digestion steps. These methodological considerations are important, as they may contribute to the differences observed between the current proteomics data and our previous Western blot results, as discussed above for Pgp and BCRP [11]. Such discrepancies highlight the limitations of both techniques in quantifying protein expression. While the limitations of targeted proteomics analysis have been discussed above, Western blot analysis may also overestimate protein levels due to antibody cross-reactivity or non-specific binding [45]. These challenges underscore the need for careful interpretation of results and the use of complementary techniques to validate findings in protein expression studies.

Of note, as the primary aim of our study was to evaluate adenoviral-infected hCMEC/D3 and hBMEC as endothelial cells to investigate the role of OATP2B1 at the BBB, this transporter was a central focus of our present analysis with the intention to supplement previous findings. However, performing targeted proteomics analysis we failed to detect OATP2B1 in hCMEC/D3 Ad-OATP2B1 and hBMEC Ad-OATP2B1. Accordingly, we supplemented the data set with an untargeted proteomic analysis, in which we were able to detect a significant increase in OATP2B1 abundance in hBMEC upon Ad-OATP2B1 infection, while no significant change was observed in hCMEC/D3. These untargeted proteomics results align with our previous study, where Western blot analysis also revealed increased OATP2B1 expression only in hBMEC following Ad-OATP2B1 infection [11].

The lack of OATP2B1 detection in the targeted proteomics approach was rather unexpected. One potential explanation for our inability to detect OATP2B1 by the selected proteotypic peptides may be due to post-translational modifications or artifactual chemical changes that occurred during sample processing [46]. In the targeted proteomics the peptides YYNNDLLR and SSPAVEQQLLVSGPGK were used for the measurement of OATP2B1 protein amount. However, performing untargeted proteomics only YYNNDLLR was detected within the group of OATP2B1 identifying peptides. These findings suggest post-translational modification of the SSPAVEQQLLVGSPGK peptide. In fact, the NetPhos predictor [47] identified a phosphorylation probability exceeding 99% for this particular peptide. The detection of YYNNDLLR exclusively in the untargeted proteomics analysis points to the fact that there were differences in the sample preparation. More in detail, the samples were dried and resuspended during untargeted proteomics preparation, likely increasing their concentration [1, 44, 48].

The elements discussed above can also be applied to explain the discrepancies found between targeted and untargeted proteomics comparison of hCMEC/D3 Ad-OATP2B1 and hBMEC Ad-OATP2B1. More in detail, evaluation by untargeted proteomics displayed higher levels of the ABC transporters Pgp and MRP1 and of several SLC transporters in hCMEC/D3 Ad-OATP2B1. Despite not being statistically significant, OATP2B1 expression was higher in hBMEC Ad-OATP2B1 when compared to hCMEC/D3 Ad-OATP2B1, which is in line with our previous comparison study [11]. In addition to the higher levels of OATP2B1 in Ad-OATP2B1 hBMEC, the untargeted proteomics analysis also revealed a change in the levels of proteins related to the cell cycle and viral infection. Despite that, the expression levels of other BBB markers, ABC, and SLC transporters remained unchanged in both cell lines, confirming that Ad-OATP2B1 increases OATP2B1 expression without altering the overall cell phenotype.

This project intended to continue the characterization and comparison of hCMEC/D3 and hBMEC for their applicability as a cell model to study transport across the BBB [11]. Endothelial cells of the BBB cultured on transwell permeable supports are assumed to exhibit polarized expression of transporters and other BBB marker proteins, closely resembling the in vivo BBB environment. This is particularly important for permeability studies and for examining the specific localization of proteins in apical and basolateral membranes. In our study we focused on providing a comprehensive proteomic profile of these cells, emphasizing overall protein abundance rather than specific localization or transport function. While culturing cells on collagen-coated transwell supports may influence the polarization of certain proteins [49, 50], previous studies have demonstrated that key BBB markers and transporters remain expressed in endothelial cells grown on standard plates, though potentially with altered polarization [1, 7]. Additionally, a limitation of this study is the lack of time-course analysis of protein expression during cell culture. Future research could benefit from investigating protein expression and polarization at different time points of cell growth and confluence, which would provide more comprehensive insights into the dynamic changes in BBB model characteristics. Nevertheless, our results indicate that hCMEC/D3 have a more solid phenotype compared to hBMEC, making them usable as brain endothelial cells to study the function of endogenously expressed transporters. However, our current and previous findings [11], along with studies from other groups [10, 51], suggest that hCMEC/D3 cells exhibit leakiness, posing a significant challenge given the need for a tight cell monolayer in cellular permeability studies. On the contrary, hBMEC showed higher tightness [10, 11] and have been previously used as a model to predict the permeability of compounds at the BBB [9].

Of note, it is important to acknowledge the limitations of our study design, particularly regarding statistical power and sample size. While our analysis detected significant changes in OATP2B1 expression, specifically in hBMEC, the absence of observed changes in some BBB markers should be interpreted cautiously. The sample size used, while sufficient to detect large effects, may have limited power to identify subtler changes in protein expression. This limitation is particularly relevant for proteins that showed no significant changes. It is possible that smaller, yet biologically relevant, alterations in these proteins might exist but were not detectable given our current experimental design. Additionally, the variability inherent in biological systems and proteomic analyses can impact the detection of small effect sizes. Therefore, while our results suggest that the adenoviral system does not broadly alter the BBB phenotype, we cannot definitively rule out all potential effects.

Although the discrepancies between targeted and untargeted proteomics underscore the importance of employing orthogonal methods to validate protein expression levels, we can still conclude that adenoviral infection can be used to selectively express transporters of interest without altering the expression of BBB markers, ABC, and SLC transporters in hBMEC. Conversely, low transduction efficiency may explain the lack of increased transporter expression following adenoviral infection observed in hCMEC/D3. As we did not assess transduction efficiency, it is plausible that variability in transporter expression across the cell population influenced the observed results. Such variability is inherent to the use of adenoviral viruses in different cells, where transduction is more efficient in less differentiated compared to differentiated cells (Meyer Zu [52]).

Based on these considerations, although with their limitations, we still suggest that both hCMEC/D3 and hBMEC can be used as a human brain endothelial cell model to study transport at the BBB. However, it is essential to select the model that best aligns with the specific requirements of the research question at hand. Doing so will enhance the validity of the findings and ensure that the results accurately reflect the phenomena being investigated.

In conclusion, our study highlights the complexities and challenges in quantifying protein expression in cellular models of the BBB. The discrepancies observed between different analytical methods emphasize the need for a multi-faceted approach in BBB research. Future studies should focus on refining proteomics techniques for membrane protein detection, exploring alternative methods for protein expression in BBB models, and further validating these in vitro models against in vivo data. Despite the identified limitations, our findings provide valuable insights into the strengths and weaknesses of altering transporter expression in BBB cell models used for in vitro transport studies.

## Supplementary Information

Below is the link to the electronic supplementary material.

Supplementary file1 (XLSX 1877 KB)

Supplementary file2 (XLSX 13 KB)

## Data Availability

The proteomic data have been submitted to the ProteomeXchange Consortium (https://www.proteomexchange.org/) through the MassIVE partner repository, using the MassIVE dataset identifier MSV000096107 and the ProteomeXchange identifier PXD056849. Additionally, the data supporting the findings of this study are available upon reasonable request from the corresponding authors.

## Abbreviations

ABC: ATP-binding cassette
BBB: Blood-brain barrier
BCECs: Brain capillary endothelial cells
CNS: Central nervous system
DDM: N-dodecyl-B-D-maltoside
FCS: Fetal calf serum
LLOQ: Lower limit of quantification
MRM: Multiple reaction monitoring
MRPs: Multidrug resistance protein
OATPs: Organic anion-transporting polypeptides
PIC: Protease inhibitor cocktail
RT: Room temperature
SLC: Solute carrier
TEAB: Triethylammonium bicarbonate
TCEP: Tris(2-carboxyethyl)phosphine hydrochloride
